# Supplementary material for: CFI-1 functions unilaterally to restrict gap junction formation in C. elegans
Source: Development. 2025 Jan 7;152(1):dev202955. doi: 10.1242/dev.202955 (PMC11829774; doi:10.1242/dev.202955)
Supplement: Supplementary information [file develop-152-202955-s1.pdf]

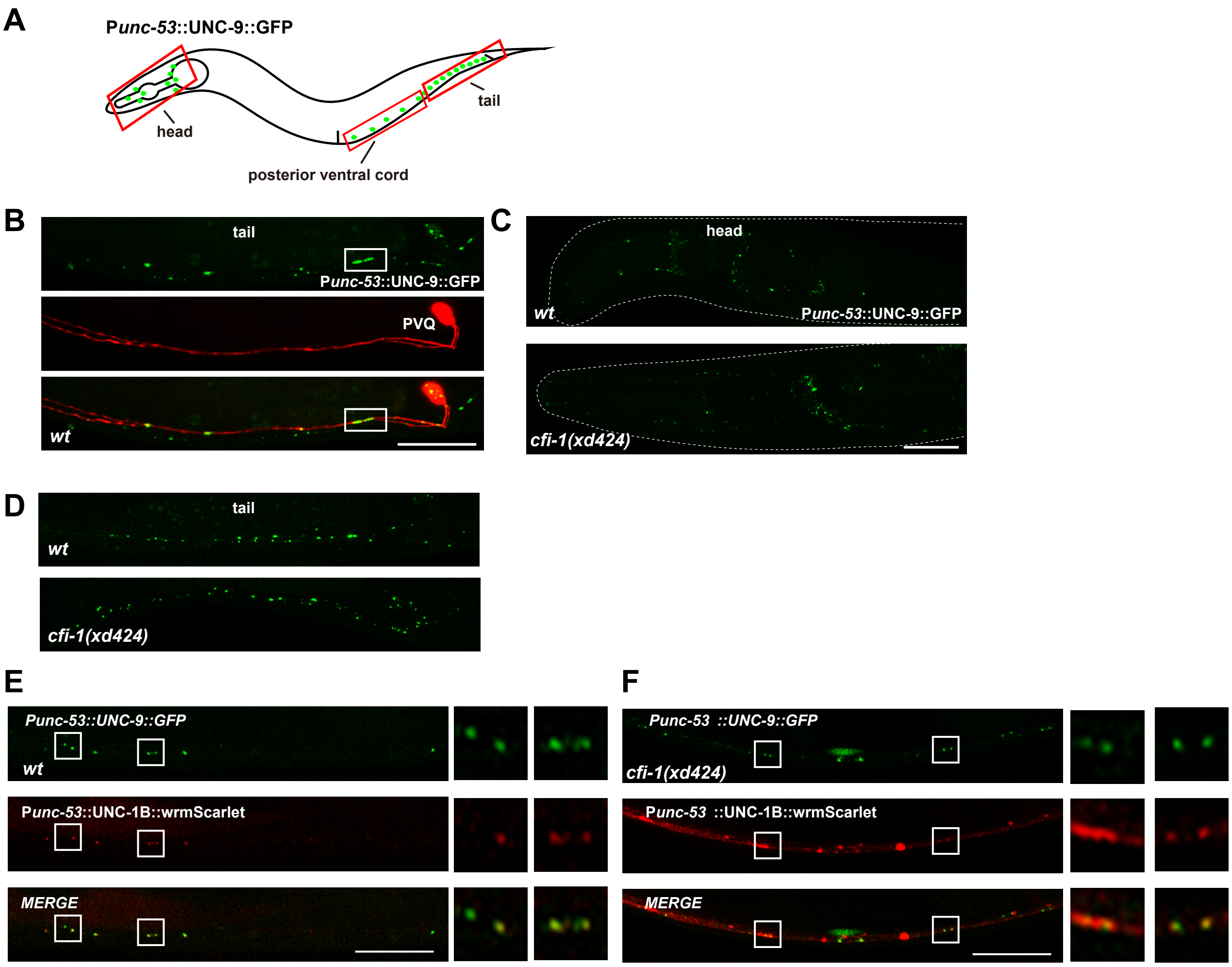

Fig. S1. Gap junction distribution of P*unc-53* expressing neurons. (A) Schematic drawing of the distribution of UNC-9::GFP puncta (green) formed by P*unc-53* expressing neurons. (B) Colocalization between P*unc-53*::UNC-9::GFP puncta and PVQ neuron (red) in the tail region. Scale bar: 25 µm. (C) P*unc-53*::UNC-9::GFP puncta in the head region in wild type (*wt*) and *cfi-1(xd424)* animals. (D) P*unc- 53*::UNC-9::GFP puncta in the tail region in wild type (*wt*) and *cfi-1(xd424)* animals. (E-F) The co-distribution P*unc-53*::UNC-1B::wrmScarlet (red) with P*unc-53*::UNC- 9::GFP (green) in wild type (*wt*) (G) and *cfi-1 (xd424)* mutant (H) animals. Scale bar: 25 µm.

Development • Supplementary information

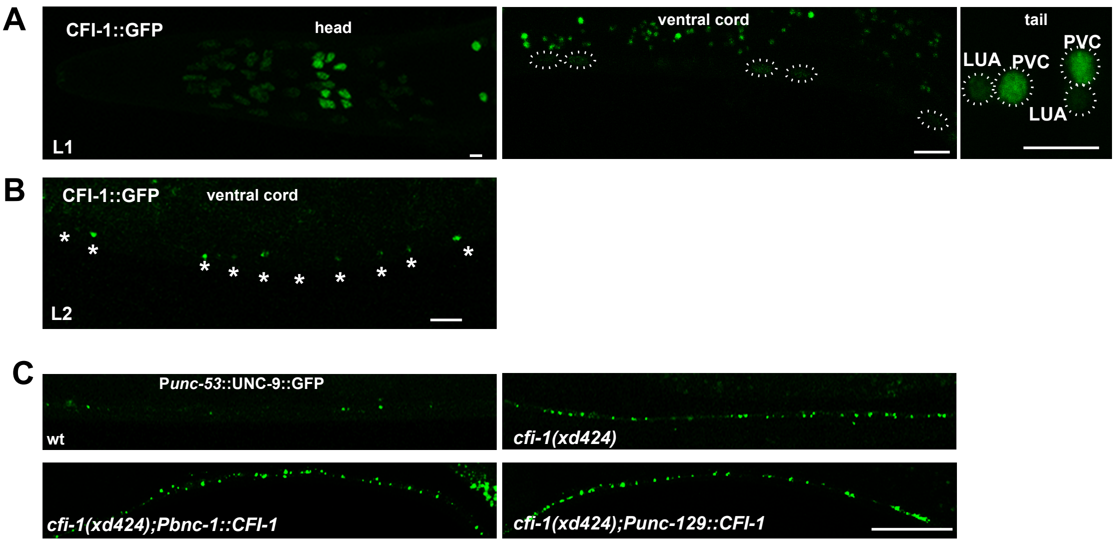

Fig. S2. The endogenous expression of *cfi-1* gene. (A and B) CFI-1::GFP knock-in (green) distribution in the head, ventral cord, and the tail region in L1 (A) and L2 (B) stage. Dashed circles and asterisks denote the cell bodies of cells expressing CFI-1::GFP. Scale bar, 10 µm. (C) *cfi-1(xd424)* mutant phenotype could not be rescued by expressing wild type copy of *cfi-1* in the ventral cord region. Scale bar: 25 µm.

Development • Supplementary information

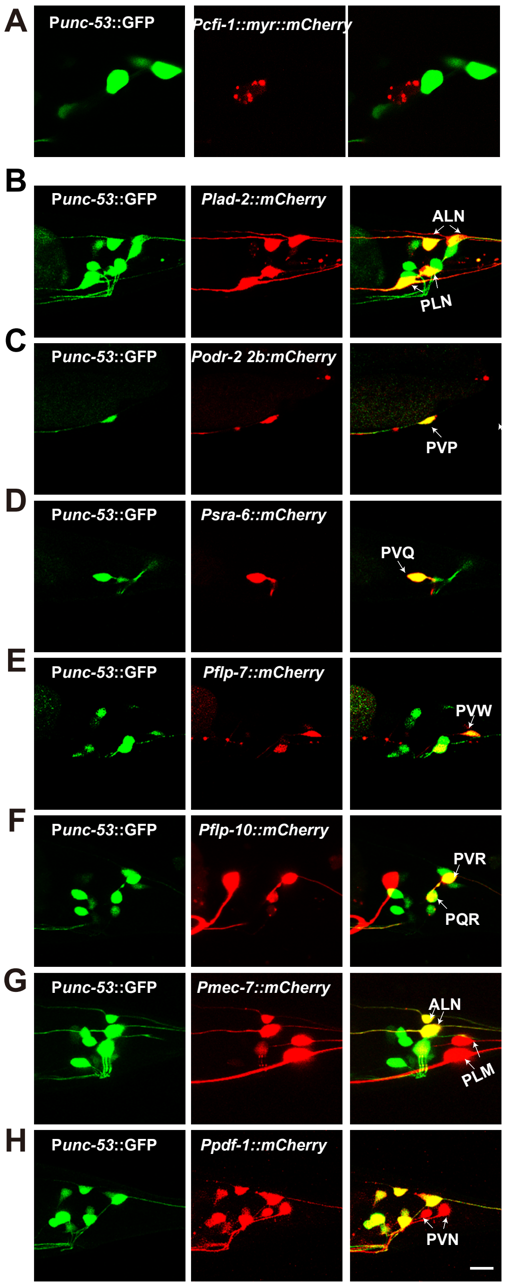

Fig. S3. Identification of the P*unc-53* expressing neurons. (A) *cfi-1* (red) (labeled by P*cfi-1*::myr::mCherry) is not expressed in P*unc-53* expressing neurons (green). (B) P*lad-2* driven mCherry labels ALN and PLN neurons (red). (C) P*odr-2 2b* driven mCherry labels PVP neurons (red). (D) P*sra-6* driven mCherry labels PVQ neurons (red). (E) P*flp-7* driven mCherry labels PVW neurons (red). (F) P*flp-10* driven mCherry labels PQR and PVR neurons (red). (G) P*mec-7* driven mCherry labels ALN and PLM neurons (red). (H) P*pdf-1* driven mCherry labels PVN neurons (red). Scale bar: 10 µm.

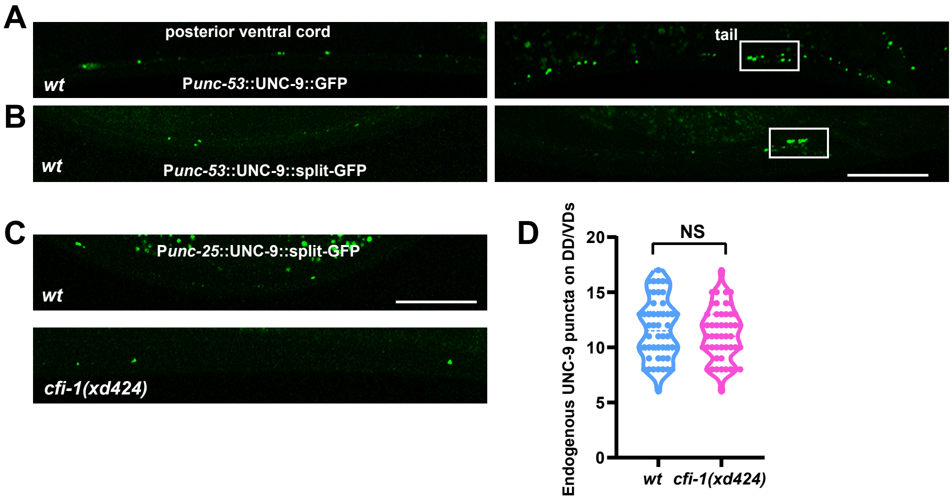

Fig. S4. The endogenous gap junction formation of DD/VD neurons is not affected by *cfi-1*. (A and B) The UNC-9::split-GFP puncta distribution resembles P*unc- 53*::UNC-9::GFP in the posterior ventral cord and tail region. (C) The distribution of endogenous gap junctions (labeled by P*unc-25*::UNC-9::split-GFP, green) in wild type (*wt*) and *cfi-1(xd424)* mutants. Scale bar: 25 µm. (D) Quantification of the P*unc- 25*::UNC-9::split-GFP puncta number in wild type (*wt*) and *cfi-1(xd424)* mutants. Student's *t*-test was performed. NS: not significant, N ≥ 50.

Development • Supplementary information

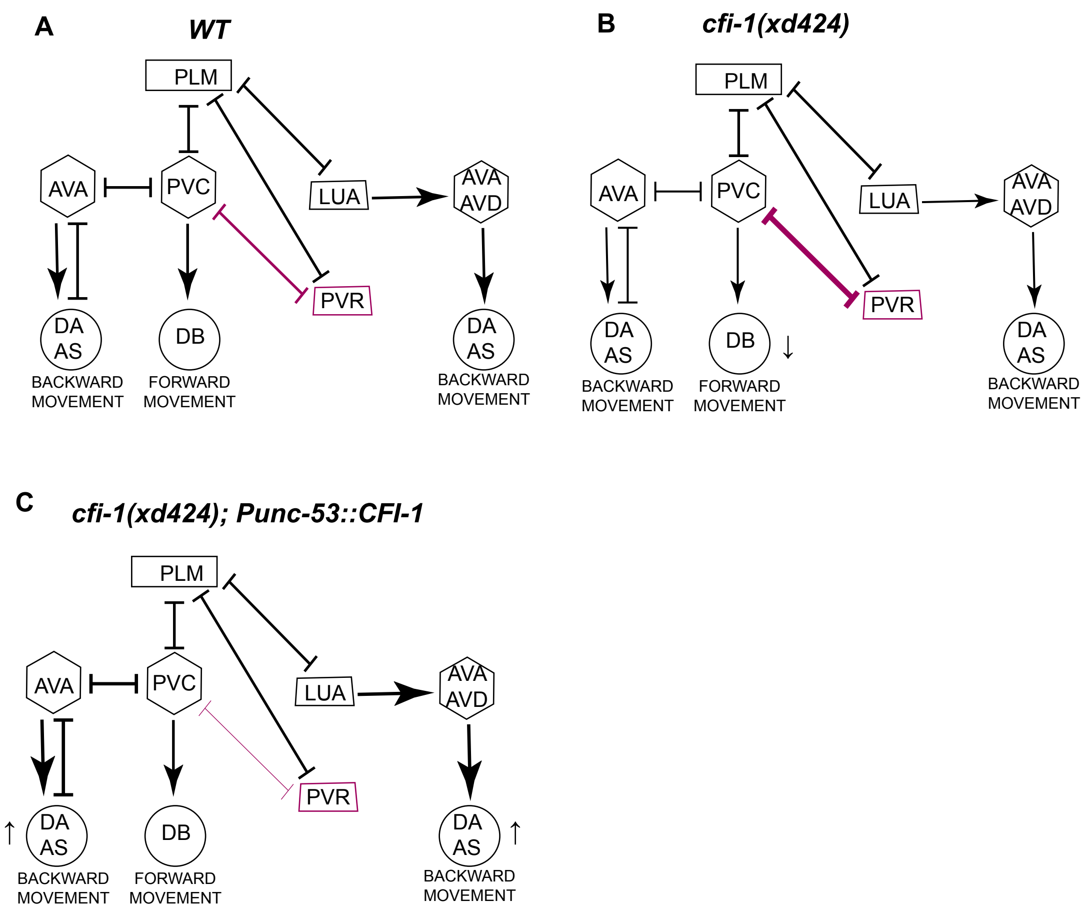

Fig. S5. Neural circuitry underlying posterior touch response. (A-C) The neural circuitry underlying posterior touch response in wild type (A), *cfi-1* mutant (B) with or without wild type *cfi-1* being expressed in PVR neuron (C). The touch cell connectors, LUA, and possible PVR, are marked by rectangles. The interneurons are marked by hexagons. The motor neurons are marked by circles. Both chemical synapses (→) and gap junctions (┤) are indicated. The PVC-PVR gap junction is highlighted (purple). The thickness of individual lines represents the different strength of signaling.
