# Supplementary material for: CFI-1 functions unilaterally to restrict gap junction formation in C. elegans
Source: Development. 2025 Jan 7;152(1):dev202955. doi: 10.1242/dev.202955 (PMC11829774; doi:10.1242/dev.202955)
Supplement: Supplementary file 1 [file develop-152-202955_review_history.pdf]

CFI-1 functions unilaterally to restrict gap junction formation in *C. elegans*

Zan Wu, Lin Pang and Mei Ding
DOI: 10.1242/dev.202955

Editor: Swathi Arur

Review timeline
Original submission: 14 April 2024
Editorial decision: 28 May 2024
First revision received: 24 September 2024
Editorial decision: 16 October 2024
Second revision received: 5 November 2024
Accepted: 8 November 2024

Original submission

First decision letter

MS ID#: DEVELOP/2024/202955

MS TITLE: CFI-1/ARID3 functions unilaterally to restrict gap junction formation in C. elegans

AUTHORS: Zan Wu, Pang Lin, and Mei Ding

I have now received all the referees' reports on the above manuscript, and have reached a decision. The referees' comments are appended below, or you can access them online: please go to BenchPress and click on the 'Manuscripts with Decisions' queue in the Author Area.

As you will see, the referees express considerable interest in your work, but have some significant suggestions and recommendations for improvement of the analysis and rigor of the study. If you are able to revise the manuscript along the lines suggested, which may involve further experiments, I will be happy to receive a revised version of the manuscript. Your revised paper will be re-reviewed by one or more of the original referees, and acceptance of your manuscript will depend on your addressing satisfactorily the reviewers' major concerns. Please also note that Development will normally permit only one round of major revision. If it would be helpful, you are welcome to contact us to discuss your revision in greater detail. Please send us a point-by-point response indicating your plans for addressing the referees' comments, and we will look over this and provide further guidance.

Please attend to all of the reviewers' comments and ensure that you clearly highlight all changes made in the revised manuscript. Please avoid using 'Tracked changes' in Word files as these are lost in PDF conversion. I should be grateful if you would also provide a point-by-point response detailing how you have dealt with the points raised by the reviewers in the 'Response to Reviewers' box. If you do not agree with any of their criticisms or suggestions please explain clearly why this is so.

Reviewer 1

Advance summary and potential significance to field

© 2025. Published by The Company of Biologists under the terms of the Creative Commons Attribution License (https://creativecommons.org/licenses/by/4.0/).

In this article, Wu et al. analyzed the role of the transcription factor CFI-1/ARID3 on gap junction formation in C. elegans. Using a genetic screen, they identified CFI-1 as a regulator of gap junction. They show that CFI-1 is expressed in the PVC neuron but not in the PVR neuron, and that loss of CFI-1 in PVC leads to an increase in gap junction numbers between PVC and PVR. Interestingly, this phenotype can be rescued by CFI-1 expression in PVC or PVR. These are interesting novel observations on the regulation of gap junction formation.

Comments for the author

Overall, the data are convincing, and the paper is well written. However, a few points could be improved before publication.
- In wild-type animals, CFI-1 is expressed in the PVC neuron but not in the PVR neuron, and there are some gap junctions between PVC and PVR. When CFI-1 is lost in PVC, the number of gap junctions between PVC and PVR is increased. This increase can be suppressed by CFI-1 expression in either PVC or PVR, leading the authors to propose a bidirectional communication between PVC and PVR in the regulation of gap junction numbers. Along these lines, it would be interesting to test the effect of an expression of CFI-1 in both PVC and PVR at the same time. This could be done by expressing CFI-1 in PVR in a wild-type background (where CFI-1 is already expressed in PVC).

- The authors have checked that, in cfi-1 mutants, Punc-53::gfp is not expressed in additional neurons. Have they also checked that, in the neurons that normally express Punc-53::gfp, the level of expression is in not increased ? This could be done by quantifying the GFP fluorescence level.

- In the result section “cfi-1 could regulate touch response from either PVR or PVC neuron”, it would be interesting to mention that it has been previously shown that a cfi-1 mutation leads to a loss of expression of some key effector genes in PVC (such as the vesicular acetylcholine transporter unc-17 or the choline transporter cho-1; Glenwinkel et al. eLife 2021), as this may affect PVC function.

- Fig. S2 legend: it would be useful to explain what is labelled with the stars on picture S2B.

Reviewer 2

Advance summary and potential significance to field

In this manuscript, the authors use C. elegans as a model to study the formation of gap junctions between PVC and PVR neurons. They found that CFI-1 functions in PVC neurons to suppress the clustering of UNC-9 puncta between PVC and PVR neurons and concluded that CFI-1 can negatively regulate gap junction formation in a cell type-specific manner. Overall, it is a very interesting study and may shed light on the translational control of gap junction formation. The genetic studies are beautifully executed, but I do have some concerns about the data interpretation.

Major concerns:
Based on the increase in UNC-9 puncta, the authors concluded that there is an increase in gap junctions between PVC and PVR neurons. While this could be the case, it is also possible that the composition rather than the quantity of gap junctions was affected by CFI-1, with more UNC-9 being assembled into and replacing other innexins in gap junctions while the number of gap junctions remains unchanged. As CFI-1 is a terminal factor for neuronal fate determination, it is likely that it will be required for the expression of some innexins in PVC neurons. Therefore, the lack of expression of certain innexins in PVC neurons could alter the composition of PVC hemichannels, consequently affecting the composition of PVR hemichannels, for example, leading to an increase in UNC-9. This could be the case in the PVR expression of CF1-1 experiences. Examination of gap junctions between PVC/PVR by electron microscopy is the most reliable way to address this concern, but I understand the difficulty of employing this technique for EM studies. I would suggest that the authors test multiple innexin markers for PVC/PVR neurons, and it may also be helpful to assess the abundance of gap junction binding proteins (for example, UNC-1) at PVC/PVR gap junctions.

© 2025. Published by The Company of Biologists under the terms of the Creative Commons Attribution License (https://creativecommons.org/licenses/by/4.0/).

Additionally, it seems that the scale bar in Figure 4H was missing.

Comments for the author

In this manuscript, the authors use C. elegans as a model to study the formation of gap junctions between PVC and PVR neurons. They found that CFI-1 functions in PVC neurons to suppress the clustering of UNC-9 puncta between PVC and PVR neurons and concluded that CFI-1 can negatively regulate gap junction formation in a cell type-specific manner. Overall, it is a very interesting study and may shed light on the translational control of gap junction formation. The genetic studies are beautifully executed, but I do have some concerns about the data interpretation.

Major concerns:
Based on the increase in UNC-9 puncta, the authors concluded that there is an increase in gap junctions between PVC and PVR neurons. While this could be the case, it is also possible that the composition rather than the quantity of gap junctions was affected by CFI-1, with more UNC-9 being assembled into and replacing other innexins in gap junctions while the number of gap junctions remains unchanged. As CFI-1 is a terminal factor for neuronal fate determination, it is likely that it will be required for the expression of some innexins in PVC neurons. Therefore, the lack of expression of certain innexins in PVC neurons could alter the composition of PVC hemichannels, consequently affecting the composition of PVR hemichannels, for example, leading to an increase in UNC-9. This could be the case in the PVR expression of CF1-1 experiences. Examination of gap junctions between PVC/PVR by electron microscopy is the most reliable way to address this concern, but I understand the difficulty of employing this technique for EM studies. I would suggest that the authors test multiple innexin markers for PVC/PVR neurons, and it may also be helpful to assess the abundance of gap junction binding proteins (for example, UNC-1) at PVC/PVR gap junctions.

Additionally, it seems that the scale bar in Figure 4H was missing.

<u>Reviewer 3</u>

Advance summary and potential significance to field

The paper addresses an interesting question, transcriptional regulation of neuron-specific placement of gap junctions, and is generally well-written (with occasional misuse of the English language that can be easily corrected).

Comments for the author

Substantive concerns:

- The authors use nmr-1::mCherry to label the PVC neuron in the tail region. Nmr-1::mCherry is also expressed in at least 3 additional neurons (AVD, AVF, AVG) that also extend processes into the region would thus be indistinguishable in their images purporting to show localization of the innexin UNC-9::GFP to PVC

- The authors propose that PVC makes gap junctions with PVR but EM reconstructions of the C. elegans L4/adult ventral nerve cord do not report gap junctions between PVC and PVR (see nemanode). It’s certainly possible that the EM reconstruction failed to detect PVC—PVR gap junctions but the authors do not address this obvious disparity with their findings. I would want to see additional evidence of this novel connection, for example, by showing co-localization of the innexin UNC-7 in PVR which is a well-established partner of UNC-9 at heterotypic gap junctions.

- Are novel UNC-9::GFP puncta actually functional gap junctions?

- Does cfi-1 control gap junction number by regulating transcription of unc-9 in PVC (or PVR)? The authors note this obvious possibility but do not test it.

- No known role for PVR in touch sensitivity.

© 2025. Published by The Company of Biologists under the terms of the Creative Commons Attribution License (https://creativecommons.org/licenses/by/4.0/).

First revision

Author response to reviewers' comments

Reviewer 1 Advance Summary and Potential Significance to Field:

In this article, Wu et al. analyzed the role of the transcription factor CFI-1/ARID3 on gap junction formation in *C. elegans*. Using a genetic screen, they identified CFI-1 as a regulator of gap junction. They show that CFI-1 is expressed in the PVC neuron but not in the PVR neuron, and that loss of CFI-1 in PVC leads to an increase in gap junction numbers between PVC and PVR. Interestingly, this phenotype can be rescued by CFI-1 expression in PVC or PVR. These are interesting novel observations on the regulation of gap junction formation.

We sincerely appreciate the reviewer's positive feedback and kind encouragement.

Reviewer 1 Comments for the Author:

Overall, the data are convincing, and the paper is well written. However, a few points could be improved before publication.

- In wild-type animals, CFI-1 is expressed in the PVC neuron but not in the PVR neuron, and there are some gap junctions between PVC and PVR. When CFI-1 is lost in PVC, the number of gap junctions between PVC and PVR is increased. This increase can be suppressed by CFI-1 expression in either PVC or PVR, leading the authors to propose a bidirectional communication between PVC and PVR in the regulation of gap junction numbers. Along these lines, it would be interesting to test the effect of an expression of CFI-1 in both PVC and PVR at the same time. This could be done by expressing CFI-1 in PVR in a wild-type background (where CFI-1 is already expressed in PVC).

Good suggestion. To test the effect of an expression of CFI-1 in both PVC and PVR at the same time, we performed two experiments. Firstly, we utilized the P*unc-53* promoter to overexpress CFI-1 specifically in PVR within wild-type animals. Our observations revealed a reduction in the number of UNC-9-positive gap junction puncta. Additionally, we employed the P*rgef-1* promoter to drive pan-neuronal expression of CFI-1, which also resulted in a decreased number of gap junction puncta. Collectively, these findings support the hypothesis that CFI-1 suppresses gap junction formation *in vivo*.

NOTE: We have removed unpublished data that had been provided for the referees in confidence

Response Figure 1. Overexpression of CFI-1 in wild type inhibits gap junction formation. (A) The P*unc-53* promoter or P*rgef-1*-driven CFI-1 expression in wild type reduced the number of UNC-9::GFP puncta (green). Scale bar: 25 μm. (B) The relative change of UNC-9::GFP puncta number in wildtype (*wt*) and *cfi-1* (overexpression) animals. One-way ANOVA with Tukey's multiple comparisons test was performed. Data are presented as mean ± s.d. ***, $P < 0.001$. $N \geq 14$ for each genotype.

- The authors have checked that, in cfi-1 mutants, Punc-53::gfp is not expressed in additional neurons. Have they also checked that, in the neurons that normally express Punc-53::gfp, the level of expression is in not increased ? This could be done by quantifying the GFP fluorescence level.

Excellent suggestion. We have quantified the P*unc-53*::GFP fluorescence intensity in both wild-type and *cfi-1(ot786)* mutant strains. Our results indicate that the expression level of P*unc-53*::GFP in the *cfi-1* mutant is not significantly elevated compared to wild-type controls. We have incorporated this data into Figure 1J for further reference.

- In the result section "cfi-1 could regulate touch response from either PVR or PVC neuron", it

© 2025. Published by The Company of Biologists under the terms of the Creative Commons Attribution License (https://creativecommons.org/licenses/by/4.0/).

would be interesting to mention that it has been previously shown that a cfi-1 mutation leads to a loss of expression of some key effector genes in PVC (such as the vesicular acetylcholine transporter unc-17 or the choline transporter cho-1; Glenwinkel et al. eLife 2021), as this may affect PVC function.
Excellent suggestion. As the reviewer correctly noted, *cfi-1* appears to influence the expression of several key effector genes, such as the vesicular acetylcholine transporter UNC-17 and the choline transporter CHO-1, both of which are critical for neuronal function in PVC. We have now incorporated this important information into the manuscript (Discussion) and have provided appropriate references.

- Fig. S2 legend: it would be useful to explain what is labelled with the stars on picture S2B.
Sorry about the oversight. We have revised the figure legend for Figure S2B to include the clarification that "asterisks denote the cell bodies of cells expressing CFI-1::GFP."

***** Reviewer 2 Advance Summary and Potential Significance to Field:

In this manuscript, the authors use C. elegans as a model to study the formation of gap junctions between PVC and PVR neurons. They found that CFI-1 functions in PVC neurons to suppress the clustering of UNC-9 puncta between PVC and PVR neurons and concluded that CFI-1 can negatively regulate gap junction formation in a cell type- specific manner. Overall, it is a very interesting study and may shed light on the translational control of gap junction formation. The genetic studies are beautifully executed, but I do have some concerns about the data interpretation.
We sincerely appreciate the reviewer's positive feedback and kind encouragement.

Major concerns:
Based on the increase in UNC-9 puncta, the authors concluded that there is an increase in gap junctions between PVC and PVR neurons. While this could be the case, it is also possible that the composition rather than the quantity of gap junctions was affected by CFI-1, with more UNC-9 being assembled into and replacing other innexins in gap junctions while the number of gap junctions remains unchanged.
We appreciate the reviewer's insightful suggestion. In addition to UNC-9, the gap junction protein UNC-7 is also broadly expressed within the worm nervous system. When we introduced the P*unc-53*::UNC-7::GFP marker into *cfi-1* mutant animals, we observed an increase in the number of UNC-7::GFP puncta. Similarly, we examined the expression of the neuronal innexin protein INX-13 and found that in *cfi-1* mutant animals, the number of Punc-53::INX-13::GFP puncta was also elevated compared to wild-type controls. These findings suggest that the *cfi-1* mutation affects the overall abundance of gap junction connections rather than specifically altering UNC-9 aggregates. We have included these data in Figures 1D-G.

As CFI-1 is a terminal factor for neuronal fate determination, it is likely that it will be required for the expression of some innexins in PVC neurons. Therefore, the lack of expression of certain innexins in PVC neurons could alter the composition of PVC hemichannels, consequently affecting the composition of PVR hemichannels, for example, leading to an increase in UNC-9. This could be the case in the PVR expression of CF1-1 experiences. Examination of gap junctions between PVC/PVR by electron microscopy is the most reliable way to address this concern, but I understand the difficulty of employing this technique for EM studies. I would suggest that the authors test multiple innexin markers for PVC/PVR neurons, and it may also be helpful to assess the abundance of gap junction binding proteins (for example, UNC-1) at PVC/PVR gap junctions.
We thank the reviewer for the excellent suggestion. We have expanded our analysis to include two additional innexin proteins, UNC-7 and INX-13. UNC-7, like UNC-9, is widely expressed throughout the worm nervous system. Upon introducing the Punc-53::UNC-7::GFP marker into *cfi-1* mutant animals, we observed an increase in the number of UNC-7::GFP puncta (Figure 1D, E). Similarly, the examination of the neuronal innexin protein INX-13 revealed an elevated number of P*unc-53*::INX-13::GFP puncta in *cfi-1* mutant animals compared to wild-type controls (Figure 1F, G). These observations suggest that the *cfi-1* mutation influences the overall abundance of gap junction connections rather than specifically altering UNC-9 aggregates. *unc-1* encodes stomatin-like protein and has been shown to regulate gap junctions in *C. elegans* (Chen et al., 2007). In wild type animals, we found that the co-expressed (P*unc-53* driven) UNC-1::wrmScarlet signal is

© 2025. Published by The Company of Biologists under the terms of the Creative Commons Attribution License (https://creativecommons.org/licenses/by/4.0/).

adjacent to UNC-9::GFP puncta. When *cfi*-1 mutant was introduced into the double labeling marker, we found that the UNC-1::wrmScarlet signal is markedly increased. Meanwhile, the UNC-9::GFP signal remains spatially adjacent to the elevated UNC-1::wrmScarlet signal (Figure S1E, F).
Collectively, these findings indicate that the *cfi-1* mutation affects the abundance of gap junction connections between PVC and PVR, rather than altering the composition of these connections.

Additionally, it seems that the scale bar in Figure 4H was missing.
Apologies for the oversight. We have included the scale bar in Figure 4H, which is now Figure 4I.

***** Reviewer 3 Advance Summary and Potential Significance to Field:

The paper addresses an interesting question, transcriptional regulation of neuron- specific placement of gap junctions, and is generally well-written (with occasional misuse of the English language that can be easily corrected).
We sincerely appreciate the reviewer's positive feedback and kind encouragement.

Reviewer 3 Comments for the Author:
Substantive concerns:
•The authors use nmr-1::mCherry to label the PVC neuron in the tail region.
Nmr-1::mCherry is also expressed in at least 3 additional neurons (AVD, AVF, AVG) that also extend processes into the region would thus be indistinguishable in their images purporting to show localization of the innexin UNC-9::GFP to PVC
We appreciate the reviewer's insightful comments and are pleased to have the opportunity to address these concerns. The P*nmr-1* promoter-driven mCherry labels additional neurons, specifically AVD, AVF, and AVG, in the head region, which also extend processes into the posterior ventral cord region where the gap junction markers were analyzed. To verify our findings, we mainly employed the following two approaches. Firstly, in our analysis involving PVC neurons, we consistently traced neurites originating from the cell body. In most instances, we were able to clearly follow the neuronal processes of PVC to the posterior ventral cord. On the rare occasions when PVC neurites could not be unambiguously traced, we excluded such data from further consideration. This approach confirms that the observed PVR gap junctions are indeed associated with PVC neurons. Second, besides PVC and LUA neurons, *cfi-1* is also expressed in AVD, URA, and IL2 neurons in the head region. URA and IL2 neurons confine their neurites to the head region, while AVD extends into the ventral nerve cord. To ascertain whether PVR gap junctions are located on AVD neurons, we utilized the P*magi-1* promoter-driven mCherry to specifically label AVD neurons (Steak et al., 2009). When co-labeled with UNC-9::GFP from PVR, we observed that the PVR gap junctions, represented by UNC-9::GFP puncta, did not co-localize with AVD neurons. In summary, our findings indicate that PVR forms gap junctions specifically with PVC neurons.

NOTE: We have removed unpublished data that had been provided for the referees in confidence

Response Figure 2. The P*unc-53* promoter driven UNC-9::GFP puncta are not situated on AVD neuron.

•The authors propose that PVC makes gap junctions with PVR but EM reconstructions of the C. elegans L4/adult ventral nerve cord do not report gap junctions between PVC and PVR (see nemanode). It's certainly possible that the EM reconstruction failed to detect PVC—PVR gap junctions but the authors do not address this obvious disparity with their findings. I would want to see additional evidence of this novel connection, for example, by showing co-localization of the innexin UNC-7 in PVR which is a well- established partner of UNC-9 at heterotypic gap junctions.
Excellent suggestion. By CeNGEN (https://cengen.shinyapps.io/CengenApp/), UNC-7 is expressed in the PVR neuron. To further verify the gap junction between PVC and PVR, we co-labeled UNC-9 in PVC and UNC-7 in PVR and found that the UNC-9::GFP puncta are tightly adjacent with UNC-7::wrmScarlet puncta. We have included these data in Figures 4H.

•Are novel UNC-9::GFP puncta actually functional gap junctions?

© 2025. Published by The Company of Biologists under the terms of the Creative Commons Attribution License (https://creativecommons.org/licenses/by/4.0/).

We appreciate the reviewer's insightful question. Electron microscopy remains the most definitive method for examining gap junctions; however, tracking specific gap junctions between individual neurons *in vivo* presents significant technical challenges. Electroporation represents another reliable approach for assessing whether the novel or additional UNC-9::GFP puncta are functional gap junctions in *cfi-1* mutants. However, performing such manipulations *in vivo*, especially within the small neurons of *C. elegans*, is also notably difficult. Instead, we have performed a series of other experiments to address this issue. Firstly, in addition to UNC-9, we also examined innexin proteins UNC-7 and INX-13. When we introduced the UNC-7::GFP marker into *cfi-1* mutant animals, we observed an increase in UNC-7::GFP puncta (Fig. 1D, E). Similarly, using the INX-13::GFP marker revealed an elevated number of INX-13::GFP puncta in *cfi-1* mutants compared to wild-type controls (Fig. 1F, G). Second, *unc-1* encodes a stomatin-like protein and has been shown to regulate gap junctions in *C. elegans* (Chen et al., 2007). In wild-type animals, we found that the UNC-1::wrmScarlet signal is adjacent to UNC-9::GFP puncta. In *cfi-1* mutants, there was a significant increase in the UNC-1::wrmScarlet signal, while the UNC-9::GFP signal remained closely associated with the elevated UNC-1::wrmScarlet signal (Fig. S1E, F). These findings indicate that the *cfi-1* mutation enhances the abundance of gap junction connections in PVR without altering their composition. Third, to further confirm the gap junctions between PVC and PVR, we co-labeled UNC-9 in PVC and UNC-7 in PVR. We observed that UNC-9::GFP puncta were tightly adjacent to UNC-7::wrmScarlet puncta, reinforcing the presence of functional gap junctions between these neurons (Fig. 4H). Fourth, gentle touch stimuli applied to the posterior region of *cfi-1* mutant animals inhibited anterior movement (Fig. 5K), while stimuli applied to the anterior region resulted in posterior locomotion similar to wild-type animals (Fig. 5J). This behavior aligns with the role of *cfi-1* in modulating electrical coupling between PVC and PVR neurons. Introducing wild-type *cfi*-1 into PVC neurons successfully rescued the posterior touch response defect in cfi-1 mutants, underscoring the critical role of PVCs in this response (Fig. 5K and Fig. S5A). Fifth, we also tested whether excessive gap junction formation between PVC and PVR neurons could be mitigated by expressing *cfi-1* in PVR neurons. Remarkably, introducing wild-type *cfi- 1* into PVR neurons also alleviated the posterior touch response defect in *cfi-1* mutants (Fig. 5K). Overall, these observations suggest that the *cfi-1* mutation leads to an excess of UNC-9::GFP puncta, which likely represent functional gap junctions between PVC and PVR.

•Does cfi-1 control gap junction number by regulating transcription of unc-9 in PVC (or PVR)? The authors note this obvious possibility but do not test it.
To address whether *cfi-1* affects the gene expression level of *unc-9*, we employed several analytical approaches. Firstly, we conducted a transcriptome analysis, which indicated that the expression level of *unc-9* is not significantly altered in *cfi-1* mutants. Subsequently, we performed quantitative reverse transcription PCR (qRT-PCR) to further investigate *unc-9* expression. Our qRT-PCR results corroborated the transcriptome data, showing no substantial changes in *unc-9* expression in *cfi-1* mutant animals compared to wild-type controls. Additionally, the *cfi-1* ChIP-seq data (Li et al., 2023) indicate that the *unc-9* gene does not appear to contain a CFI-1 binding site. *cfi-1* is expressed in diverse motor neurons within the ventral cord. However, the overall distribution of gap junctions on ventral cord motor neurons does not appear to be affected by *cfi-1* mutations. This highlights the cell-context dependent nature of *cfi-1* in regulating gap junctions. Therefore, if *cfi-1* does influence *unc*-9 expression, any effects are likely confined to a very limited number of cells.

•No known role for PVR in touch sensitivity.
We appreciate the reviewer's insightful observation. While PVR does not appear to play an essential role in touch sensitivity itself, its distinct neuronal connections suggest a role in modulating the touch circuitry. PVR forms gap junctions with ALM and PLM neurons, which are crucial for the anterior touch response. PVR also synapses onto RIP neurons. Notably, ablation of RIP results in an unresponsive pharyngeal pumping to light touch (Avery and Thomas, 1997; Chalfie et al., 1985; Albertson and Thomson, 1976). Additionally, PVR forms gap junctions with the LUA neuron, which is identified as a touch cell connector in the posterior touch movement circuit (Chalfie et al., 1985; White et al., 1986). Our study further demonstrates that PVR also forms gap junctions with PVC neurons, which are essential for posterior touch response. These findings suggest that by attenuating neuronal activity in associated networks through its gap junction connections, PVR may act more as a modifier of touch responses rather than as a direct executor of those responses.

© 2025. Published by The Company of Biologists under the terms of the Creative Commons Attribution License (https://creativecommons.org/licenses/by/4.0/).

References
Albertson, D.G., Thomson, J.N. (1976) The pharynx of *Caenorhabditis elegans*. *Philos. Trans. R. Soc. Lond. B. Biol. Sci.* **275**, 299-325. doi: 10.1098/rstb.1976.0085
Avery, L., Thomas, J.H. (1997). Feeding and defecation. In: Riddle DL, Blumenthal T, Meyer BJ, Priess JR, Riddle DL, Blumenthal T, Meyer BJ, Priess JR, editors. *C. elegans II. Cold Spring Harbor, NY: Cold Spring Harbor Laboratory Press,* p 679-716.
Chalfie, M., Sulston, J. E., White, J. G., Southgate, E., Thomson, J. N. and Brenner, S. (1985) The neural circuit for touch sensitivity in *Caenorhabditis elegans*. *J. Neurosci.* **5**, 956-964. doi: 10.1523/JNEUROSCI.05-04-00956.1985
Chen, B., Liu, Q., Ge, Q., Xie, J., and Wang, Z.W. (2007). UNC-1 regulates gap junctions important to locomotion in *C. elegans*. *Curr. Biol.* **17**, 1334-1339. doi: 10.1016/j.cub.2007.06.060
Li, Y., Smith, J. J., Marques, F., Osuma, A., Huang, H. C. and Kratsios, P. (2023). Cell context-dependent CFI-1/ARID3 functions control neuronal terminal differentiation. *Cell Rep.* **42**, 112220. doi: 10.1016/j.celrep.2023.112220
Stetak, A., Horndli, F., Maricq, A.V., van den Heuvel, S., and Hajnal, A. (2009). Neuron-specific regulation of associative learning and memory by MAGI-1 in *C. elegans*. PLoS One **4**, e6019. doi: 10.1371/journal.pone.0006019
White, J. G., Southgate, E., Thomson, J. N. and Brenner, S. (1986) The structure of the nervous system of *Caenorhabditis elegans*. *Philos. Trans. R. Soc. Lond. B. Biol. Sci.* **314**, 1-340. doi: 10.1098/rstb.1986.0056

Second decision letter

MS ID#: DEVELOP/2024/202955

MS TITLE: CFI-1/ARID3 functions unilaterally to restrict gap junction formation in C. elegans

AUTHORS: Zan Wu, Lin Pang, and Mei Ding

I have now received all the referees reports on the above manuscript, and have reached a decision. The referees' comments are appended below, or you can access them online: please go to BenchPress and click on the 'Manuscripts with Decisions' queue in the Author Area.

The overall evaluation is positive and we would like to publish a revised manuscript in Development. However, as suggested by reviewer 3, please cite the Starch et al, study from 2009, and add the textual edits. While I do not expect to send the paper back to the reviewers, I will look at it. Thus, I ask that you carefully provide point by point response. If you do not agree with any of their criticisms or suggestions explain clearly why this is so. If it would be helpful, you are welcome to contact us to discuss your revision in greater detail. Please send us a point-by-point response indicating your plans for addressing the referees' comments, and we will look over this and provide further guidance.

Reviewer 1

Advance summary and potential significance to field

Revised version of the manuscript.

Comments for the author

The authors have addressed in a satisfactory manner all the points that I raised.

Reviewer 2

© 2025. Published by The Company of Biologists under the terms of the Creative Commons Attribution License (https://creativecommons.org/licenses/by/4.0/).

Advance summary and potential significance to field

In this manuscript, the authors use C. elegans as a model to study the formation of gap junctions between PVC and PVR neurons. They found that CFI-1 functions in PVC neurons to suppress the clustering of UNC-9 puncta between PVC and PVR neurons, concluding that CFI-1 can negatively regulate gap junction formation in a cell type-specific manner. This is a very interesting study that sheds light on the translational control of gap junction formation.

Comments for the author

The authors have addressed all my concerns. Congratulations!

<u>Reviewer 3</u>

Advance summary and potential significance to field

Regulation of gap junction assembly by a conserved transcription factor

Comments for the author

The authors have satisfactorily addressed my concerns with the exception of the question of whether cfi-1 regulates expression of unc-9/innexin:
The central finding of this paper is that the transcription factor CFI-1/ARID3 limits the number of apparent gap junction plaques between two specific pairs of C. elegans neurons, PVC and PVR; gap junction puncta at the PVC/PVR interface are elevated in cfi-1 loss-of-function mutants. Gap junction puncta are visualized with the innexin UNC-9::GFP. In the discussion, the authors acknowledge the possibility that cfi-1 regulates (i.e., represses) transcription of unc-9 and potentially other gap junction-associated proteins that are similarly affected in cfi-1 mutants. The authors note in response to my review that they have conducted "transcriptional analysis" and qRT-PCR that did not detect elevation of unc-9 in cfi-1 mutant animals. (These data are not included in the manuscript) From this limited description, however, it's difficult to assess the significance of these findings. For example, unc-9 is likely expressed in every C. elegans neuron so whole animal transcriptomics, if that was the approach used here, would not detect a change in unc-9 expression in a small subset of neurons. On the other hand, if the authors conducted cell-specific transcriptomics from isolated PVC neurons, then these data would be convincing and should be included in the paper. Otherwise, a transcriptional reporter knockin at the unc-9 locus or smFISH for the native unc-9 transcript both scored in PVC neurons could resolve this question. Or, the authors can opt not to perform these experiments. But, I think this is an obvious missed opportunity that could be resolved with available technology.

Minor revisions:
This paper (Starich et al. 2009) should be cited since it was the first to provide direct evidence of heterotypic UNC-7/UNC-9 gap junctions and that the assembly of each of these innexins depended on the presence of the other in the partner neuron.

"…had not been unidentified." should be "…had not been identified."

"Next, we went to identify with which punc-53 expressing neurons the PVCs form gap junctions" could be rewritten to say: "Next, we set out to identify the punc-53-expressing neurons with which the PVD neurons make gap junctions."

"…critical role in gap junction assembling." Should be "critical role in gap junction assembly."

"…regulates the gene expression of cfi-1…" should be "regulates expression of cfi-1…"

What does this mean? "…the electrically coupled neuron pair is not an inseparable unit."

© 2025. Published by The Company of Biologists under the terms of the Creative Commons Attribution License (https://creativecommons.org/licenses/by/4.0/).

Starich TA, Xu J, Skerrett IM, Nicholson BJ, Shaw JE. 2009. Interactions between innexins UNC-7 and UNC-9 mediate electrical synapse specificity in the Caenorhabditis elegans locomotory nervous system. Neural Develop. 4(1):16.

Second revision

Author response to reviewers' comments

Point-to-point response:

Reviewer 1 Comments for the Author:

The authors have addressed in a satisfactory manner all the points that I raised.
We sincerely thank this reviewer for the instructive comments and suggestions.

***** Reviewer 2 Advance Summary and Potential Significance to Field:

In this manuscript, the authors use C. elegans as a model to study the formation of gap junctions between PVC and PVR neurons. They found that CFI-1 functions in PVC neurons to suppress the clustering of UNC-9 puncta between PVC and PVR neurons, concluding that CFI-1 can negatively regulate gap junction formation in a cell type- specific manner. This is a very interesting study that sheds light on the translational control of gap junction formation.
Reviewer 2 Comments for the Author:
The authors have addressed all my concerns. Congratulations!
We sincerely thank this reviewer for the encouragement and instructive suggestions.

***** Reviewer 3 Advance Summary and Potential Significance to Field:

Regulation of gap junction assembly by a conserved transcription factor Reviewer 3 Comments for the Author: The authors have satisfactorily addressed my concerns with the exception of the question of whether cfi-1 regulates expression of unc-9/innexin. The central finding of this paper is that the transcription factor CFI- 1/ARID3 limits the number of apparent gap junction plaques between two specific pairs of C. elegans neurons, PVC and PVR; gap junction puncta at the PVC/PVR interface are elevated in cfi-1 loss-of-function mutants. Gap junction puncta are visualized with the innexin UNC-9::GFP. In the discussion, the authors acknowledge the possibility that cfi-1 regulates (i.e., represses) transcription of unc-9 and potentially other gap junction-associated proteins that are similarly affected in cfi-1 mutants. The authors note in response to my review that they have conducted “transcriptional analysis” and qRT-PCR that did not detect elevation of unc-9 in cfi-1 mutant animals. (These data are not included in the manuscript) From this limited description, however, it’s difficult to assess the significance of these findings. For example, unc-9 is likely expressed in every C. elegans neuron so whole animal transcriptomics, if that was the approach used here, would not detect a change in unc-9 expression in a small subset of neurons. On the other hand, if the authors conducted cell-specific transcriptomics from isolated PVC neurons, then these data would be convincing and should be included in the paper. Otherwise, a transcriptional reporter knockin at the unc-9 locus or smFISH for the native unc-9 transcript both scored in PVC neurons could resolve this question. Or, the authors can opt not to perform these experiments. But I think this is an obvious missed opportunity that could be resolved with available technology.

We sincerely thank this reviewer for the encouragement and instructive suggestions. The reviewer rightly pointed out that cell-specific transcriptomics, particularly from isolated cell populations, has proven to be a valuable approach for investigating whether gene expression is selectively altered in specific subsets of cells. Additionally, methods such as transcriptional reporter knock-ins at native gene loci or single-molecule RNA fluorescence in situ hybridization (smFISH) would indeed be effective techniques for addressing the issue at hand. We fully acknowledge the merit of the

© 2025. Published by The Company of Biologists under the terms of the Creative Commons Attribution License (https://creativecommons.org/licenses/by/4.0/).

reviewer's suggestions and agree that the proposed experiments are highly valuable. However, we believe there are several important considerations that warrant further clarification. First, it is essential to note that, while overexpression of either connexins or innexins in cultured cells is sufficient to drive the assembly of gap junctions between random pairs of adjacent cells, gap junctions do not form between adjacent neurons *in vivo*, even when these cells express compatible gap junction subunits. This suggests that simple regulation of gap junction protein transcripts may not fully account for the mechanisms underlying gap junction formation *in vivo*. Moreover, many gap junction proteins have notably short half-lives compared to other transmembrane proteins, which implies that their synthesis, ER-to-Golgi trafficking, recycling, and degradation must also be tightly regulated processes. Furthermore, in the nervous system, the precise subcellular localization of gap junctions must be carefully controlled. Therefore, while we fully recognize the value of the reviewer's proposed experiments, we believe that a more comprehensive investigation is required to understand how *cfi-1* specifically regulates gap junctions in the PVC. Additionally, we would like to highlight that the three experiments suggested by the reviewer, while insightful, are technically challenging, time-consuming, and may not always yield reliable results. The complexity of these approaches introduces considerable experimental variability, which could complicate the interpretation of results. Given these factors, we believe that incorporating these experiments into the current manuscript may not be feasible at this time. Furthermore, while the scientific questions raised by these experiments are certainly important, they extend beyond the core focus of the present study and would be more appropriately addressed in a future, more focused investigation.

Minor revisions:

This paper (Starich et al. 2009) should be cited since it was the first to provide direct evidence of heterotypic UNC-7/UNC-9 gap junctions and that the assembly of each of these innexins depended on the presence of the other in the partner neuron.
We have cited the paper in the manuscript.

"…had not been unidentified." should be "…had not been identified."
The sentence in the manuscript has been corrected.

"Next, we went to identify with which punc-53 expressing neurons the PVCs form gap junctions" could be rewritten to say: "Next, we set out to identify the punc-53- expressing neurons with which the PVD neurons make gap junctions."
We have revised the sentence in the manuscript.

"…critical role in gap junction assembling." Should be "critical role in gap junction assembly."
We have revised the sentence in the manuscript.

"…regulates the gene expression of cfi-1…" should be "regulates expression of cfi-1…"
We have revised the sentence in the manuscript.

What does this mean? "…the electrically coupled neuron pair is not an inseparable unit."
We have revised the sentence to: "Intriguingly, the expression of *cfi-1* in PVR neurons, but not in PVC neurons, leads to abnormal backward movement in response to posterior touch, indicating that, despite their electrical coupling, PVC and PVR neurons do not function as a unified entity."

Third decision letter

MS ID#: DEVELOP/2024/202955

MS TITLE: CFI-1/ARID3 functions unilaterally to restrict gap junction formation in C. elegans

AUTHORS: Zan Wu, Lin Pang, and Mei Ding
ARTICLE TYPE: Research Article

© 2025. Published by The Company of Biologists under the terms of the Creative Commons Attribution License (https://creativecommons.org/licenses/by/4.0/).

I am happy to tell you that your manuscript has been accepted for publication in Development, pending our standard publication integrity checks.

© 2025. Published by The Company of Biologists under the terms of the Creative Commons Attribution License (https://creativecommons.org/licenses/by/4.0/).
